# Clinical and Immunological Features of SARS-CoV-2 Breakthrough Infections in Vaccinated Individuals Requiring Hospitalization

**DOI:** 10.1007/s10875-022-01325-2

**Published:** 2022-07-09

**Authors:** Giulia Lamacchia, Alessio Mazzoni, Michele Spinicci, Anna Vanni, Lorenzo Salvati, Benedetta Peruzzi, Sara Bencini, Manuela Capone, Alberto Carnasciali, Parham Farahvachi, Arianna Rocca, Seble Tekle Kiros, Lucia Graziani, Lorenzo Zammarchi, Jessica Mencarini, Maria Grazia Colao, Roberto Caporale, Francesco Liotta, Lorenzo Cosmi, Gian Maria Rossolini, Alessandro Bartoloni, Laura Maggi, Francesco Annunziato

**Affiliations:** 1 Department of Experimental and Clinical Medicine, University of Florence, Viale Pieraccini, 6, 50134 Florence, Italy; 2 Infectious and Tropical Diseases Unit, Careggi University Hospital, Florence, Italy; 3 Flow Cytometry Diagnostic Center and Immunotherapy, Careggi University Hospital, Florence, Italy; 4 Microbiology and Virology Unit, Careggi University Hospital, Florence, Italy; 5 Immunology and Cell Therapy Unit, Careggi University Hospital, Florence, Italy

**Keywords:** COVID-19, Vaccine, Autoantibodies, SARS-CoV-2, T-cell, B-cell

## Abstract

**Background and Purpose:**

Waning immunity and the surge of SARS-CoV-2 variants are responsible for breakthrough infections, i.e., infections in fully vaccinated individuals. Although the majority of vaccinated infected subjects report mild or no symptoms, some others require hospitalization. The clinical and immunological features of vaccinated hospitalized COVID-19 patients are currently unknown.

**Methods:**

Twenty-nine unvaccinated and 36 vaccinated hospitalized COVID-19 patients were prospectively enrolled and clinical and laboratory data were gathered. Immunophenotyping of leukocytes’ subsets, T and B cell SARS-CoV-2-specific responses were evaluated via flow cytometry. Anti-IFN-α autoantibodies were measured via ELISA.

**Results:**

Despite vaccinated patients were older and with more comorbidities, unvaccinated subjects showed higher levels of pro-inflammatory markers, more severe disease, and increased mortality rate. Accordingly, they presented significant alterations in the circulating leukocyte composition, typical of severe COVID-19. Vaccinated patients displayed higher levels of anti-Spike IgGs and Spike-specific B cells. Of all participants, survivors showed higher levels of anti-Spike IgGs and Spike-specific CD4+ T cells than non-survivors. At hospital admission, 6 out of 65 patients (9.2%) displayed high serum concentrations of autoantibodies targeting IFN-α. Remarkably, 3 were unvaccinated and eventually died, while the other 3 were vaccinated and survived.

**Conclusion:**

Despite more severe pre-existing clinical conditions, vaccinated patients have good outcome. A rapid activation of anti-SARS-CoV-2-specific immunity is fundamental for the resolution of the infection. Therefore, prior immunization through vaccination provides a significant contribution to prevention of disease worsening and can even overcome the presence of high-risk factors (i.e., older age, comorbidities, anti-IFN-α autoantibodies).

**Supplementary Information:**

The online version contains supplementary material available at 10.1007/s10875-022-01325-2.

## Introduction

As of the beginning of May 2022, registered COVID-19 cases have exceeded 513 million cases worldwide and more than 6.2 million fatalities [1]. Vaccines’ release in the last weeks of December 2020 changed dramatically the perspective of the ongoing COVID-19 pandemic. As vaccination campaigns proceed towards the vast immunization in most countries, one of the main open matters regards the maintenance of vaccine efficacy over time. Indeed, the progressive waning of antibody titers and the emergence of new viral variants with increased transmissibility and immune escape capability have led to increased numbers of breakthrough infections, i.e., SARS-CoV-2 infections in fully vaccinated individuals [2–4]. Published data have demonstrated efficient establishment of humoral response and cellular immunological memory induced by vaccination [5–8] as well as progressive loss of vaccine efficacy against infection 6 months following the second dose administration, although maintaining substantially conserved efficacy against severe disease [9, 10]. These observations were obtained concomitantly with the global spread of Delta variant. A third vaccine injection was demonstrated to be safe and effective in restoring vaccine efficacy [11–13]. However, the recent emergence of Omicron variant with enhanced immune escape potential [14] has been associated to increased numbers of re-infections and breakthrough infections even after booster administration [4].

Nonetheless, among the many subjects fully vaccinated who test positive for SARS-CoV-2, the striking majority reports mild or no symptoms [3]. Indeed, if the reduced antibody neutralization capability predisposes to infection, T cell immunity is substantially conserved against viral variants, thus conferring protection from severe disease [7, 15]. However, in some cases, hospitalization is needed, but the predisposing factors for increased risk of severe COVID-19 in vaccinated subjects are currently unknown. Indeed, head-to-head clinical and laboratory in-depth characterizations of vaccinated and unvaccinated hospitalized COVID-19 patients are still missing. This type of analysis may better elucidate the role of vaccination in modifying the disease course. This is of importance, as it could inform about the susceptibility of fully vaccinated subjects to SARS-CoV-2 infection or re-infection, and might also direct attention to the need for updated vaccines, highly specific for the new emerging viral strains.

In the present study, we collected clinical, laboratory, and immunological data to characterize vaccinated and unvaccinated subjects infected during the Delta variant wave in Italy, who needed hospital admission. Our results showed that, despite more severe pre-existing clinical conditions, vaccinated subjects presented a more favorable disease course, with higher survival rate than the unvaccinated patients. This clinical observation was supported by immunological findings demonstrating a pattern of significantly altered immune homeostasis in unvaccinated patients. Finally, we found that vaccination provided a rapid activation of anti-SARS-CoV-2 immunity upon infection resulting in a significant positive impact on patients’ survival.

## Materials and Methods

### Patients

COVID-19 patients admitted from November to mid-December 2021 were enrolled at the Careggi University Hospital (Azienda Ospedaliero-Universitaria Careggi), Florence, Italy, by the Infective and Tropical Diseases Unit. All COVID-19 patients requiring hospitalization were included, regardless of the time from disease onset and severity. Among these, 36 patients were fully vaccinated with any approved viral vector or mRNA SARS-CoV-2 vaccine and some of them received a booster dose (4/36), while 29 were unvaccinated. SARS-CoV-2 infection was confirmed by routine diagnostic PCR amplification of viral genes from nasopharyngeal swabs. The mean time from the last vaccine dose to infection (first positive nasopharyngeal swab) was 139 ± 63 days. All patients were treated following national and international guidelines on COVID-19 treatment according to disease severity, comorbidities, eligibility to antiviral monoclonal antibodies, and tolerability according to the latest version of the World Health Organization (WHO) living guidance for clinical management of COVID-19 [16]. Data on treatment are reported in Supplemental Table S1. Peripheral blood (PB) was collected at hospital admission in EDTA tubes for mononuclear cells (PBMC) recovery, or in clot-activator tubes for serum collection. The clinical and demographic details of each patient can be found in Supplemental Table 1.

### Evaluation of Clinical Parameters

Patients’ clinical conditions were classified on the basis of the Charlson Comorbidity Index, a method of classifying comorbid conditions that predicts mortality [17]. According to the classification of COVID-19 disease defined by WHO, we scored 0 asymptomatic, 1 mild, 2 moderate, 3 severe, and 4 critical disease [16].

SARS-CoV-2 infection and viral load were determined via nasopharyngeal swab and real-time PCR.

Evaluation of WBC count was performed using a XN 550 hematology analyzer (Sysmex Corporation, Kobe, Japan). Serum concentration of C-reactive protein (CRP), ferritin, lactate dehydrogenase (LDH), and interleukin-6 (IL-6) was performed in a Cobas analyzer (Roche Diagnostics, Penzberg, Germany). D-dimer and fibrinogen were measured in a ACLTOP550 system (Instrumentation Laboratory, Werfen Group, Kirchheim bei Munchen, Germany). All these parameters were evaluated on blood specimens collected at the same time of those for flow cytometric analysis.

### Myeloid Cell Immunophenotyping by Flow Cytometry

A stain-lyse-and-then-wash procedure was used to stain surface markers using 100 µl of whole blood. Antibodies used for flow cytometry analysis are listed in Supplemental Table 2. Data acquisition was performed using a 3-laser, 8-color flow cytometer (FACSCanto TMII, BD Biosciences, San Jose, CA) and then analyzed by Infinicyt software (Cytognos SL, Salamanca, Spain). 10^5^ total leucocytes were acquired for each analysis.

### Lymphoid Cell Immunophenotyping by Flow Cytometry

PBMCs were obtained after density gradient centrifugation of blood samples. After 2 washes in PBS, cells were stained for 15 minutes with fluorochrome-conjugated monoclonal antibodies (mAbs). Full list of all fluorochrome-conjugated mAbs is available in Supplemental Tables 3 and 4. Samples were acquired on a BD LSR II flow cytometer (BD Biosciences) and analyzed with FlowJo v10 software. All flow cytometric analyses were performed following published guidelines [18].

### Evaluation of SARS-CoV-2-Specific IgM and IgG

Evaluation of anti-Spike protein (in trimeric form) IgG (Diasorin); anti-Spike protein IgM (Abbott), anti-Nucleoprotein IgG (Abbott), neutralizing antibodies which block binding of Spike protein with the ACE2 receptor (Dia.Pro Diagnostic Bioprobes) was performed following manufacturers’ instructions. The antibody reactivity of each specimen was expressed in BAU/ml, or by the ratio between optical density and cut-off value (index).

### Spike-Specific B cells and Plasmablast Evaluation by Flow Cytometry

For Spike-specific B cells and plasmablast evaluation, 2 million PBMCs were stained for 30 minutes at 4 °C with fluorochrome-conjugated antibodies listed in Supplemental Tables 5 and 6. Recombinant biotinylated SARS-Cov2 Spike protein (Miltenyi Biotech) was conjugated separately with streptavidin PE and PE-Vio770 for 15 min at room temperature and pooled in 1:2 ratio, before being added to final staining mix. After the incubation, cells were washed with PBS/EDTA buffer (PEB), and incubated 5 minutes with 7-AAD for viability evaluation. Samples were acquired on a BD LSR II flow cytometer (BD Biosciences). Full gating strategy is reported in Supplemental Fig. 6.

### Evaluation of Polyclonal- and SARS-CoV-2-Specific T Cell Cytokine Production by Flow Cytometry

For T cell stimulation in vitro, 1.5 million PBMCs were cultured in complete RPMI plus 5% human AB serum in 96-well flat bottom plates. Cultures were performed in medium alone (background, negative control), with a pool of Spike SARS-CoV-2 peptide pools (Prot_S1, Prot_S + , and Prot_S to achieve a complete sequence coverage of the Spike protein), or with a pool of peptide pools covering nucleoprotein and membrane protein. Each peptide pool was used at 0.6 µM/peptide, accordingly to manufacturer’s instructions (Miltenyi Biotech). In addition, staphylococcal enterotoxin B SEB 1 µg/ml (Sigma-Aldrich) was used for the evaluation of polyclonal cytokine production ability. After 2 hours of incubation at 37 °C, 5% CO2, Brefeldin A (5 µg/mL) was added, followed by additional 4-hour incubation. Finally, cells were fixed and stained using fluorochrome-conjugated antibodies listed in Supplemental Table 7. Samples were acquired on a BD LSR II flow cytometer (BD Biosciences). Full gating strategy is reported in Supplemental Fig. 7. Flow cytometry experiments were performed following published guidelines [18].

### Measurement of Human Anti-IFN-α Antibodies

Titers of anti-IFN-α antibodies were measured via enzyme-linked immunosorbent assay (Invitrogen) on patients’ sera, according to manufacturer’s instructions. Sera samples of 15 young (< 40 years), sex-matched, healthy unvaccinated patients were used as negative controls.

### Statistics

Unpaired, non-parametric, Mann–Whitney’s *U* test and a *χ*^2^ test were used for comparison of clinical laboratory findings and for flow cytometric analysis of vaccinated versus unvaccinated COVID-19 patients. *P* values equal or less than 0.05 were considered significant.

## Results

### Clinical Features of Vaccinated and Unvaccinated COVID-19 Patients at Hospital Admission and Throughout Hospitalization

A total of 65 patients affected by COVID-19 and admitted to the Careggi University Hospital (Florence, Italy) were enrolled from November to mid-December 2021, a period of time that coincided with the outbreak of B1.617.2 Variant of Concern, commonly known as Delta variant [19]. The cohort was divided into the group of vaccinated subjects (*n* = 36), who received a complete vaccination cycle of any approved viral vector or mRNA SARS-CoV-2 vaccine and possibly a booster dose, and the group of unvaccinated subjects (*n* = 29). All patients tested positive for reverse transcription real-time PCR performed on nasopharyngeal swabs, confirming SARS-CoV-2 infection. Notably, the viral load was comparable between the two cohorts (Supplemental Fig. 1). Gender distribution was similar in both groups. As for the vaccinated patients, the mean age was 73 years, significantly higher (*p* < 0.05) than unvaccinated individuals’ (67 years), as shown in Supplemental Table 1. All subjects reported approximately 1 week between the onset of symptoms and hospital admission, with no differences between the two groups (Supplemental Table 1).

**Fig. 1:**
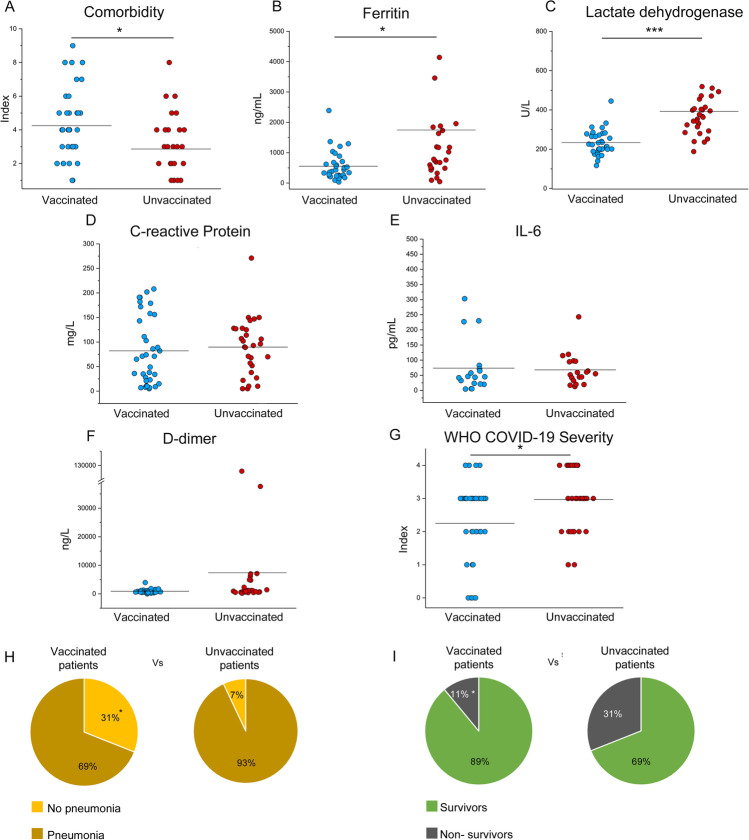
Clinical features of vaccinated and unvaccinated COVID-19 patients requiring hospitalization. Comorbidity index (**A**), serum titers of ferritin (**B**), lactate dehydrogenase (**C**), C-reactive protein (**D**), interleukin-6 (**E**), D-dimer (**F**), and WHO COVID-19 severity index of 36 vaccinated (blue dots) and 29 unvaccinated (red dots) hospitalized COVID-19 patients (**G**). (**H**) Percentages of vaccinated and unvaccinated COVID-19 hospitalized patients who developed pneumonia, on a total of 65 patients. (**I**) Percentages of vaccinated and unvaccinated COVID-19 hospitalized patients who deceased, on a total of 65 patients. Mean values are represented as black lines. *P < 0.05 and ***P < 0.0005 calculated with Mann–Whitney *U* test and *χ*^2^ test

At hospital admission, the two cohorts presented comparable pulmonary dysfunction as assessed by PaO_2_/FiO_2_ ratio (Supplemental Table 1). The Charlson Comorbidity Index was significantly higher (mean 4.3, *p* < 0.05) in the vaccinated group when compared to the unvaccinated one (mean 2.9), indicating more comorbidities among vaccinated than unvaccinated COVID-19 patients (Fig. 1A). Remarkably, laboratory tests on hospital admission revealed a significant increase of the levels of serum ferritin (*p* < 0.05) as well as of lactate dehydrogenase (*p* < 0.0005) in the unvaccinated subjects compared to the vaccinated ones (Fig. 1B–C). On the contrary, no significant differences were found in the levels of other inflammatory markers (C-reactive protein, IL-6, and D-dimer) between the two groups (Fig. 1D–F). Other demographic and clinical features, including treatment and type of vaccine administered, are summarized in Supplemental Table 1.

The severity of disease was evaluated in the course of hospitalization, and significant differences were observed between the two groups. According to a scale based on WHO disease severity classification criteria including 0 (asymptomatic), 1 (mild), 2 (moderate), 3 (severe), and 4 (critical), unvaccinated COVID-19 patients presented significantly higher disease severity index (*p* < 0.05) than vaccinated patients (Fig. 1G). Consistently, 93% (27/29) of the unvaccinated group developed pneumonia, versus 69% (24/36) of the vaccinated patients (Fig. 1H). Considering the outcome, mortality rate was 31% (9/29) in the unvaccinated cohort, while 11% (4/36) among the vaccinated group (Fig. 1I). No correlation was found between the outcome and pharmaceutical treatment or type of SARS-CoV-2 vaccine administered to enrolled patients. All survived subjects were discharged approximately 9 days after hospital admission (Supplemental Table 1). Comprehensively, these data indicated more severe disease in unvaccinated patients compared to vaccinated subjects.

### Immune Cell Landscape Reveals Marked Alterations in Unvaccinated Patients

**Fig. 2:**
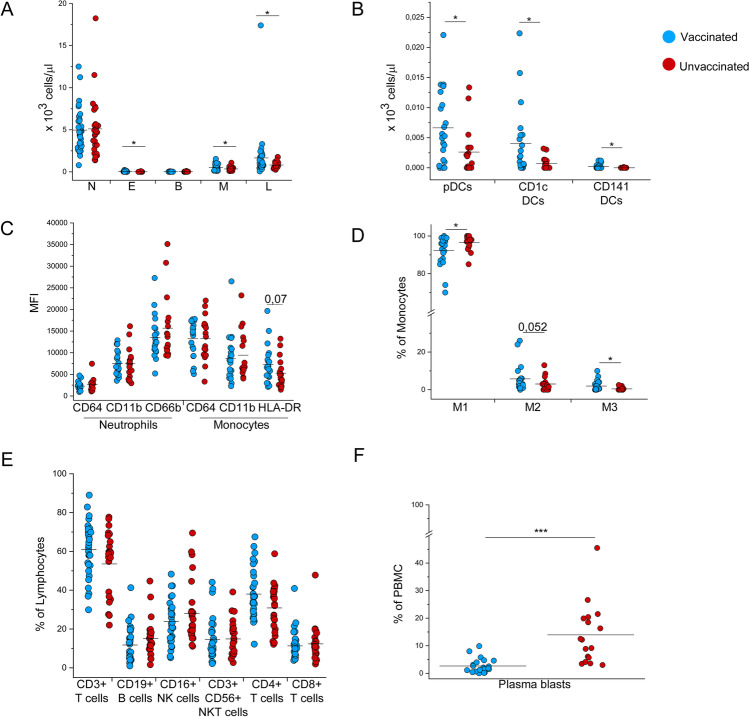
Characterization of circulating immune cell subsets in vaccinated and unvaccinated COVID-19 patients requiring hospitalization. (**A**) Absolute numbers of neutrophils (N), eosinophils (E), basophils (B), monocytes (M), and lymphocytes (L) in 36 vaccinated (blue dots) and 29 unvaccinated (red dots) COVID-19 patients requiring hospitalization. **(B)** Absolute numbers of plasmacytoid dendritic cells (pDCs), CD1c + dendritic cells and CD141 dendritic cells in 22 vaccinated (blue dots) and 17 unvaccinated (red dots) COVID-19 patients requiring hospitalization. (**C**) Mean Fluorescence Intensity of neutrophil activation markers (CD64, CD11b, and CD66b) and monocyte activation markers (CD64, CD11b, and HLA-DR) in 22 vaccinated (blue dots) and 17 unvaccinated (red dots) COVID-19 patients requiring hospitalization. (**D**) Frequency of CD14++ CD16- classical monocytes (M1), CD14 + CD16 + intermediate monocytes (M2), and CD14+ CD16++ non-classical monocytes (M3) in vaccinated (blue dots) and unvaccinated (red dots) COVID-19 patients requiring hospitalization. (**E**) Frequency of CD3+ T cells, CD19+ B cells, CD16+ NK cells, CD3+ CD56+ NKT cells, CD4+ T cells, and CD8+ T cells in 30 vaccinated (blue dots) and 26 unvaccinated (red dots) COVID-19 patients. (**F**) Frequency of plasmablasts in vaccinated (blue dots) and unvaccinated (red dots) COVID-19 patients requiring hospitalization. Black lines indicate mean values. *P < 0.05, ***P < 0.001 calculated with Mann–Whitney *U* test

Severe SARS-CoV-2 infection has a profound impact on the cells of the immune system. With the aim of producing a general characterization of the immunological profile of vaccinated and unvaccinated hospitalized individuals diagnosed with COVID-19, we evaluated leukocyte subsets’ distribution and features at hospital admission. Despite the two groups were found similar in terms of absolute numbers of white blood cells (WBC), specific subsets displayed significant differences. While neutrophils and basophils presented similar levels in both cohorts, eosinophils, monocytes, and lymphocytes’ absolute numbers were significantly reduced (*p* < 0.05) in the unvaccinated with respect to the vaccinated group (Fig. 2A). Following the same trend, the unvaccinated patients showed decreased absolute numbers of both circulating CD141 + dendritic cells (cDC1 DCs) and CD1c + (cDC2) DCs, as well as plasmacytoid DCs (pDCs) (Fig. 2B). Neutrophil activation markers such as CD11b, CD64, and CD66b were also found to be expressed at similar levels in both groups (Fig. 2C). The monocyte compartment was further characterized: CD64 and CD11b expression levels resulted similar between the two groups, while HLA-DR showed a trend towards reduced expression in the unvaccinated cohort (Fig. 2C). In addition, we dissected the proportions of classical, intermediate, and non-classical subsets. Interestingly, we found significantly lower frequencies (*p* < 0.05) of CD14+ /CD16++ (non-classical, M3) monocytes in the unvaccinated patients than in vaccinated subjects, while the opposite trend applied for CD14++ /CD16- (classical, M1) monocytes, which presented significantly higher frequencies (*p* < 0.05) in the unvaccinated group. On the contrary, the CD14++ /CD16+ (intermediate, M2) monocyte subset’s frequencies were comparable between the two cohorts (Fig. 2D).

Regarding lymphocyte subsets, no significant differences were detected in the main subpopulations, including CD3+ T cells, CD19+ B cells, and CD16+ NK cells. Among CD3+ T cells, the two cohorts exhibited comparable frequencies of CD4+ , CD8+ T cells, and CD56+ NKT cells (Fig. 2E). Further exploring the immune cell composition, we remarkably found a significant increase (*p* < 0.0005) in the frequency of circulating plasmablasts in unvaccinated compared to vaccinated COVID-19 patients (Fig. 2F). Gating strategies for the identification of myeloid and lymphoid cell subsets are reported in Supplemental Figs. 2 and 3.

We further characterized CD4+ and CD8+ T cells’ subsets to define naive and memory subpopulations (Supplemental Fig. 4). While no differences were detected in the context of CD4+ T cells, the CD8+ naive compartment showed significantly higher frequencies (*p* < 0.05) in the unvaccinated than the vaccinated group (Supplemental Fig. 5A–B). Similar expression of CXCR3 and CCR6 among CD4+ T cells, as markers of Th1 and Th17 cell polarization, as well as in CXCR5 expression, as marker of T follicular helper cell phenotype, was found in both groups (Supplemental Fig. 5C–D). Finally, since we and others showed that T cell function is significantly impaired in COVID-19 patients [20–22], we evaluated the basic functionality of the T cell compartment by studying the production of cytokines with anti-viral activity (i.e., IFN-γ, TNF-α, and IL-2) after stimulating peripheral blood mononucleated cells with superantigen Staphylococcal Enterotoxin B (SEB). Concerning the CD4+ T cell subset, we also evaluated the expression of activation surface marker CD154. Results of the stimulation showed significantly reduced levels of IL-2 (*p* < 0.005) and TNF-α (*p* < 0.05) production in the unvaccinated CD4+ T cell compartment, while no differences were found in the levels of CD154 expression and IFN-γ production (Supplemental Fig. 5E). As for CD8+ T cells, we observed an overall comparable cytokine production between vaccinated and unvaccinated patients (Supplemental Fig. 5FB). We also explored T cell polyfunctionality, as T cells that are capable of more than one function are superior in terms of antiviral activity [20, 21]. Notably, the percentage of CD4+ T cells exhibiting two to four functions (CD154+ , IL-2+ , TNF-α, IFN-γ) was significantly reduced (*p* < 0.05) in the unvaccinated group, while no differences concerning polyfunctionality were observed in the CD8+ compartment (Supplemental Fig. 5G–H).

### Vaccinated Patients Show a Higher Spike-Specific Humoral and B cell Response than Unvaccinated Patients

**Fig. 3:**
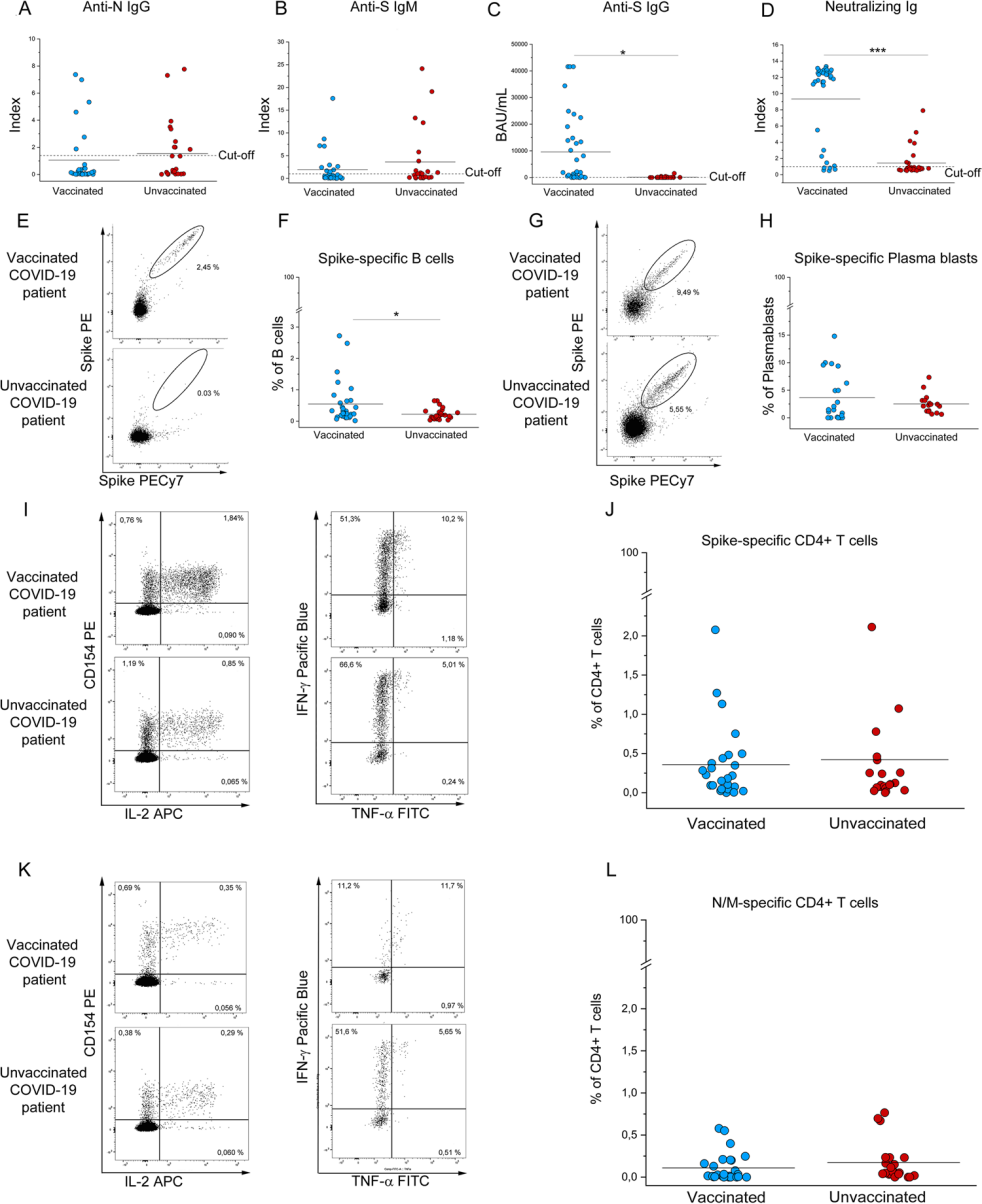
Evaluation of SARS-CoV-2-specific humoral response and circulating CD4 + T cells in vaccinated and unvaccinated COVID-19 patients requiring hospitalization. Anti-N IgG (**A**), anti-S IgM (**B**), anti-S IgG (**C**), and neutralizing Ig (**D**) titers in vaccinated (blue dots) and unvaccinated (red dots) COVID-19 patients requiring hospitalization. (**E–F**) Representative plots and frequency of Spike-specific B cells in 30 vaccinated (blue dots) and 26 unvaccinated (red dots) COVID-19 patients and Spike-specific plasmablasts (**G–H**) in 22 vaccinated (blue dots) and 18 unvaccinated (red dots) COVID-19 patients requiring hospitalization. Representative plots and frequency of Spike-specific (**I**–**J**) and N/M-specific (**K–L**) CD4+ T cells defined by CD154+ expression and production of at least 1 cytokine among IL-2, IFN-γ, and TNF-α in 26 vaccinated (blue dots) and 22 unvaccinated (red dots) COVID-19 patients requiring hospitalization. Black lines indicate mean values. *P < 0.05; ***P < 0.001 calculated with Mann–Whitney *U* test

It is conceivable that SARS-CoV-2 breakthrough infections requiring hospitalization may be the result of a weak immune response to vaccination or waning immunity. For this reason, we tested humoral and cellular anti-SARS-CoV-2 immunity in vaccinated and unvaccinated COVID-19 patients at hospital admission. Regarding antibody levels, while anti-Nucleoprotein (N) IgG and anti-Spike (S) IgM titers resulted comparable in the two groups (Fig. 3A–B), the levels of anti-S IgGs and neutralizing Ig were strikingly higher (*p* < 0.0005) in the vaccinated than in the unvaccinated subjects (Fig. 3C–D). In agreement with this result, flow cytometric analysis of S-specific B cells showed significantly higher frequencies (*p* < 0.05) in the vaccinated COVID-19 patients’ group than the unvaccinated counterpart (Fig. 3E–F). Evaluation of surface markers CD38 and CD27 coupled with BCR S-specific binding was used to study the frequencies of S-specific plasmablasts, which did not display significant differences between the two groups of patients (Fig. 3G–H). Moving to the characterization of T cell response to SARS-CoV-2, we identified by flow cytometry CD4+ T cells reactive to S or N and Membrane protein (M) by stimulating patients’ PBMCs with specific peptide pools. Reactive T cells were defined on the basis of CD154 expression and production of at least one cytokine between IL-2, TNF-α, and IFN-γ. Notably, our results showed that the S-specific and M plus N-specific CD4+ T cell response was comparable between the two patients’ groups (Fig. 3I–L).

### Deceased Patients Show Reduced Anti-Spike Immunity at Hospital Admission

**Fig. 4:**
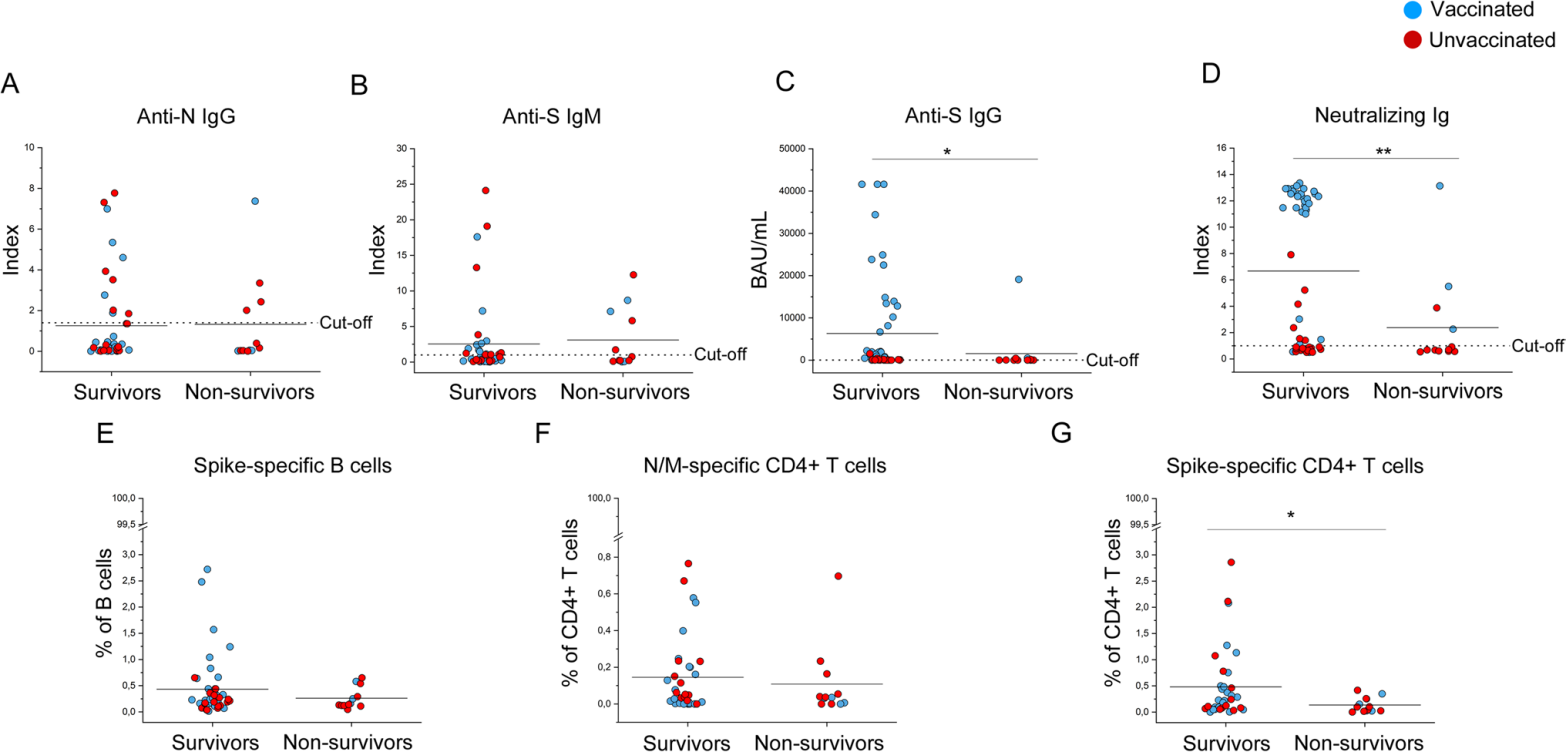
Assessment of SARS-CoV-2 specific humoral and cellular response in survived and deceased COVID-19 patients. (**A**) Titers of anti-NIgGs and (**B**) anti-S IgMs in (*n* = 44) survivors and (*n* = 12) non-survivors, titers of (**C**) anti-S IgGs and (**D**) neutralizing antibodies in (*n* = 52) survivors and (*n* = 13) non-survivors among COVID-19 hospitalized patients. (**E**) Frequency of Spike-specific B cells in (*n* = 44) survivors and (*n* = 12) non-survivors among COVID-19 hospitalized patients. (**F**) Frequency of N and M-specific CD4 + T cells in (*n* = 35) survivors and (*n* = 12) non-survivors among COVID-19 hospitalized patients. (**G**) Frequency of S-specific CD4 + T cells in (*n* = 35) survivors and (*n* = 13) non-survivors among COVID-19 hospitalized patients. Blue and red dots correspond to vaccinated and unvaccinated individuals, respectively. Black lines indicate mean values. *P < 0.05; **P < 0.01 calculated with Mann–Whitney *U* test

**Fig. 5:**
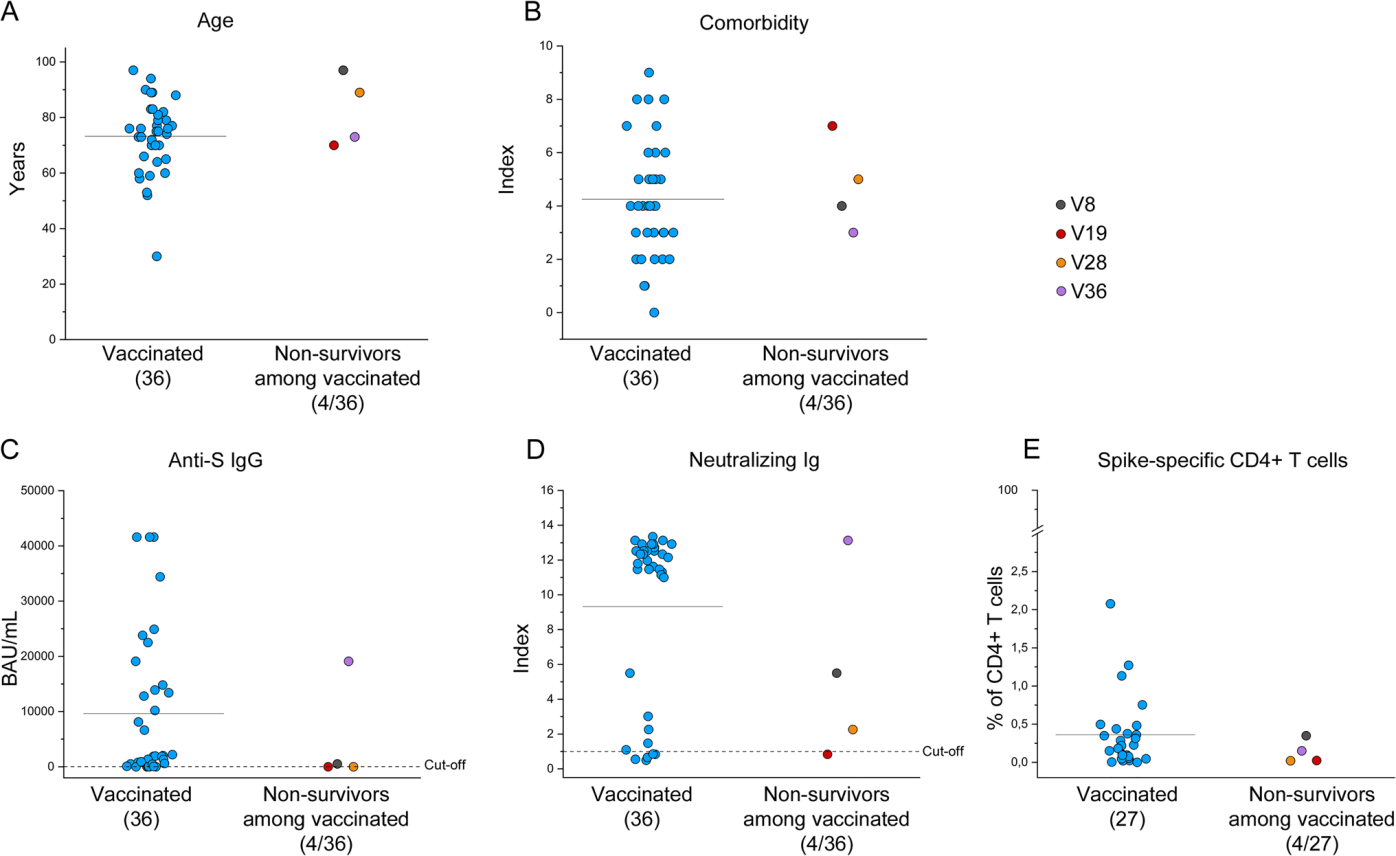
Clinical features and Spike-specific immune response of non-survivors among vaccinated patients. Mean age (**A**) and comorbidity index (**B**) of the 36 vaccinated patients (blue dots) and 4 non-survivors among the vaccinated group (multicolor dots). (**C**–**D**) Anti-S IgG and neutralizing Ig titers of vaccinated patients (blue dots) and non-survivors among the vaccinated group (multicolor dots). (**E**) Frequency of S-specific CD154+ producing at least 1 cytokine among IL-2, IFN-γ, and TNF-α among CD4 + T cells in 27 vaccinated patients (blue dots) and 4 non-survivors among the vaccinated group (multicolor dots). Black lines indicate mean values

In order to find possible predictive values of patients’ outcome, we compared anti-SARS-CoV-2 immunity at hospital admission between survivors (52/65) and non-survivors (13/65), regardless of vaccination status (nevertheless vaccinated and unvaccinated individuals are indicated with different colors in Fig. 4). Anti-N IgGs and anti-S IgMs did not differ between the two groups (Fig. 4A–B), while anti-S IgGs and neutralizing Ig were significantly higher (*p* < 0.05) in the group of survivors compared to non-survivors (Fig. 4C–D). As for S-specific B cells, no statistically significant differences were observed between the two cohorts (Fig. 4E). Regarding T cell response, CD4+ T cells reactive to N or M showed comparable frequencies in survivors and non-survivors, while S-specific CD4+ T cells showed a significantly lower frequency (*p* < 0.05) in the latter (Fig. 4F–G). When considering only vaccinated patients, among the 4 non-survivors, 2 were older than the average of the whole group (72 years) (Fig. 5A). Notably, the youngest vaccinated non-survivor (V19) displayed a higher comorbidity index than the average of the group (Fig. 5B). Non-survivors V8, V19, and V28 displayed reduced levels of anti-S IgGs and neutralizing Ig than the complete vaccinated cohort (Fig. 5C–D). Finally, V8, V28, and V36 exhibited a lower S-specific CD4 + T cell response than the average of all vaccinees (Fig. 5E). Concerning the non-survivors within the unvaccinated subjects, age and comorbidity mean index were lower compared to non-survivors in the vaccinated group (respectively, 73 years and 3,45), but higher if compared with unvaccinated survivors.

### Vaccination Can Prevent Fatal Outcome in Patients with High Anti-IFN-α Autoantibodies

**Fig. 6:**
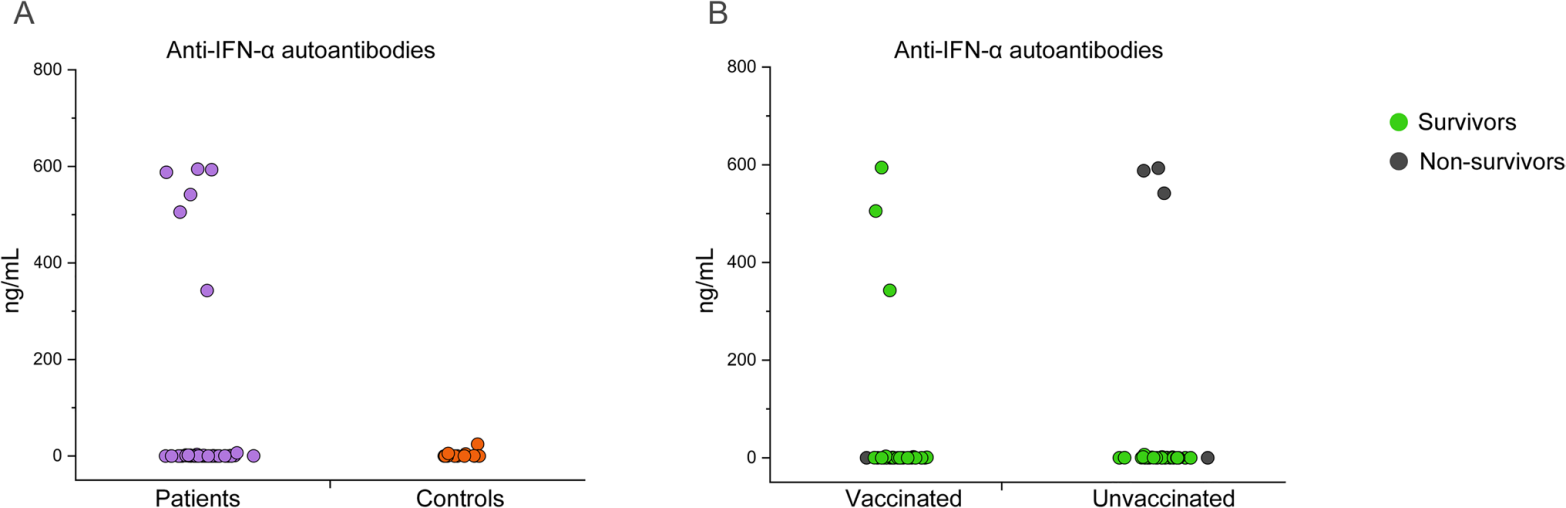
Detection of anti-IFN-α antibodies in COVID-19 patients and healthy controls. Titers of autoantibodies targeting IFN-α in all COVID-19 patients (n=65) participating to the study (violet dots) and control healthy donors (n=15) (orange dots). (B) Titers of autoantibodies targeting IFN-α in vaccinated (n=36) and unvaccinated (n=29) COVID-19 patients, marked basing on positive outcome (green dots) and negative outcome (dark gray dots)

Since it has been demonstrated that autoantibodies targeting type I IFNs predispose to severe COVID-19 development [23, 24], we measured the concentration of anti-IFN-α antibodies in all the study participants. As a control, we enrolled 15 young (< 40 years), sex-matched, healthy subjects. We found that 6 out of 65 patients (9.2%) exhibited high anti-IFN-α autoantibodies titers (Fig. 6A). Notably, 5 of these 6 patients were male, confirming previous observations showing that anti-type I IFNs antibodies occur more frequently in males [23]. Mean age of the 6 patients with high anti-IFN-α antibodies was 79 years, while the rest of the cohort displayed a mean age of 70 years (*p* = 0.07, Mann–Whitney *U* test). Of note, the only female subject among the 6 with high anti-IFN-α autoantibodies was the eldest (90 years old). Regarding the vaccination status, anti-IFN-α autoantibody–positive patients were equally divided between the vaccinated and the unvaccinated groups (3/36, 8% vs 3/29, 10% respectively) (Fig. 6B). Remarkably, taking into account the outcome, all vaccinated patients with autoantibodies targeting IFN-α were discharged, while all those unvaccinated deceased (Fig. 6B).

## Discussion

SARS-CoV-2 vaccines represented a turning point in the COVID-19 pandemic; however, as we move forward with global vaccination campaigns, many unanswered questions need to be addressed. Clinical trials and real-world studies reported high vaccine efficacy against both disease and infection [25–28]. Nonetheless, the progressive waning of antibody levels over time, together with the emergence of new viral variants with increased transmissibility and immune escape potential, led to increased breakthrough infections [3, 4]. This phenomenon has been associated to a significant surge in the numbers of vaccinated, SARS-CoV-2-infected subjects requiring hospitalization [2, 3, 12], but it is currently unknown whether disease progression in hospitalized vaccinated patients mirrors that of unvaccinated patients or differences exist between the two groups. For this reason, we performed a head-to-head comparison of clinical, laboratory, and immunological features of vaccinated and unvaccinated COVID-19 patients, who were prospectively enrolled between November and mid-December 2021, during the Delta wave in Italy [19]. Our data showed that the cohort of vaccinated subjects displayed more risk factors for severe COVID-19 development than unvaccinated patients, including older age and more comorbidities. At hospital admission, despite comparable respiratory function, unvaccinated patients showed significantly higher levels of ferritin and LDH. Since the first descriptions of COVID-19 cases, high serum ferritin levels were associated with severe and critical disease and finally deemed as a prognostic marker of COVID-19 [29–32]. Moreover, the cell-death marker LDH is a hallmark of severe COVID-19 [33], and has been correlated to respiratory failure and ARDS development [34]. The two cohorts displayed similar viral loads, suggesting that initial viral replication is not restrained by previous immunization. The disease course was also remarkably different between the two groups, as the unvaccinated cohort showed a higher frequency of patients developing pneumonia, with an overall higher COVID-19 disease severity score. More importantly, we observed a significantly higher rate of non-survivors in the unvaccinated than the vaccinated group. Altogether, our data suggest that, despite being older and affected by more comorbidities, vaccinated subjects have a substantially more favorable disease course than unvaccinated subjects.

Previous studies have shown that severe COVID-19 has a profound impact on the immune system [20, 35]. For this reason, in this study, we also investigated the main features of innate and adaptive immunity in our cohorts. Unvaccinated patients displayed reduced absolute numbers of circulating eosinophils, monocytes, and lymphocytes, as well as reduced numbers of CD1c+ and CD141+ myeloid DC as well as of pDCs. These observations are consistent with previous findings showing that in severe COVID-19, there is selective depletion of these cell subsets [20, 36]. Indeed, lymphopenia is one of the hallmarks reflecting disease severity [37]. Focusing on monocyte composition, we observed that unvaccinated patients had a lower frequency of circulating inflammatory (non-classical, M3) monocytes, with a parallel increase in the classical (M1) subset. Comparably, this pattern of monocyte redistribution has been previously demonstrated in severe patients [36] and has been associated to the selective transmigration of non-classical monocytes to inflamed lungs [38]. Regarding the lymphoid compartment, we did not find major abnormalities in the main populations of circulating cells, nor in the composition of naïve and memory T cell subsets. Notably, we found significantly higher frequencies of circulating plasmablasts in the unvaccinated cohort. Massive egress of plasmablasts in the circulation has been previously described in the context of other acute infections, including Dengue, Hepatitis A (HAV), influenza, and respiratory syncytial virus [39–41]. This observation is consistent with data from another group [42] and suggests that also in the context of SARS-CoV-2 infection, there is a massive release of antibody-secreting cells early after the infection, and it correlates with disease severity. It is currently debated if plasmablast response is driven mainly by antigen-specific cells, or if bystander cells are also involved [40, 42]. Our data showing that only a minor fraction of total circulating plasmablasts is S-specific are in agreement with those observed in the context of HAV infection, although obtained with a different experimental approach. However, it should be noted that we monitored the specificity towards only one viral antigen, thus underestimating the total SARS-CoV-2-specific plasmablast response.

Severe COVID-19 patients are characterized by an altered T cell functionality, with reduced production of cytokines with anti-viral activity [20, 43]. This impairment can be the result of an excessive stimulation by pro-inflammatory cytokines, although it remains to be formally proven. In agreement with the increased disease severity of unvaccinated patients, CD4+ T cells from these patients showed reduced production of IL-2 and TNF-α, and reduced percentages of polyfunctional CD4+ T cells than the vaccinated counterpart after stimulation with a superantigen. Regarding SARS-CoV-2-specific immunity, we found that anti-S IgGs and S-specific B cells were significantly higher in vaccinated patients, confirming that hospitalization can also occur in patients that responded to the vaccine. Anti-S IgMs and anti-N IgGs were comparable between the two cohorts, and below cut-off values in most of the patients. This finding is in agreement with the recent and comparable onset of the disease in the two groups. Moreover, this observation further supports that the difference in the levels of anti-S B cells and IgGs in the two cohorts are related to vaccination. Surprisingly, both S- and N plus M-specific CD4+ T cells showed comparable frequencies in the two cohorts. It should be noted T cells were isolated the day of patients’ hospital admission, approximately 9 days after the onset of symptoms. Therefore, it is likely that priming and reactivation of SARS-CoV2-specific T cells, respectively in the unvaccinated and the vaccinated group, produced a comparable result in the two cohorts at this time point from the infection. In agreement, it has previously been demonstrated that a rapid T cell response occurs in patients with favorable outcome [44]. For this reason, we also recast the criteria to divide our cohort and compared survivors versus non-survivors, regardless of their vaccination status. Anti-S IgGs were significantly higher in survivors, as well as S-specific B cells, despite not reaching statistical significance. Importantly, the frequency of S-specific CD4+ T cells was significantly higher in survivors versus non-survivors. Of note, when considering only vaccinated patients, we found that a weak vaccine response may contribute to the worst outcome in this group, together with pre-existing conditions including age and comorbidities, which likely were determinant for the poor outcome of non-survivors in the unvaccinated group, as already observed in past COVID-19 characterizations in pre-vaccination era [20, 30, 31]. The percentage of deceased patients being lower in the vaccinated group and including the very eldest and fragile patients reinforces the protective role of the vaccination. Previous data have demonstrated that an impaired type I IFN response, due to inborn errors or autoantibodies, is a risk factor for severe COVID-19 occurrence [23, 24, 45]. In our study, 9.2% of COVID-19 hospitalized patients exhibited high anti-IFN-α levels, a frequency that is approximately consistent with the one observed by Bastard and colleagues when measuring anti-IFN-α neutralizing antibody titers in a much larger cohort of critical and severe patients. Here, we found that among COVID-19 hospitalized patients with autoantibodies targeting IFN-α, those who were vaccinated survived, while the unvaccinated died. Also in line with previous data, anti-IFN-α autoantibody–positive patients in our cohort were predominantly males (5/6) and in older age groups; the mean age was 82 years for vaccinated patients, while it was 75 years for the unvaccinated. This also matches the age window within which the general population is found with about ten times higher auto-antibodies’ titers than individuals below 70 years of age, as reported by Bastard and colleagues, who tested several thousands of non-infected patients from all age groups. Although our data were obtained on a relatively small cohort, this observation further strengthens the concept that patients with auto-antibodies targeting not only IFN-α, but also the other cytokines of the type I IFN group, as suggested in previous works [45], are at risk to develop severe COVID-19. Furthermore, Bastard and colleagues have very recently described autoantibodies neutralizing type I IFNs in 10 (out of 42) individuals who received 2 doses of mRNA vaccine and later developed severe or critical COVID-19 [46]. The mean age of these vaccinated patients was 75 years, they were predominantly males, and remarkably all of them survived to COVID-19 [46]. Accordingly, our results emphasize the relevance of vaccination, as it can substantially protect patients with anti-type I IFNs autoantibodies.

## Conclusions

In conclusion, our data showed that vaccinated hospitalized COVID-19 patients, most likely infected with the Delta variant, displayed a better disease progression than unvaccinated patients in spite of the unfavorable pre-existing clinical conditions. The clinical observations obtained on unvaccinated patients were confirmed by an immunological landscape that reflected the one previously described in severe COVID-19 patients.

Collectively, these data confirm that a rapid activation of anti-SARS-CoV-2 immunity is mandatory to guarantee patients’ survival. This observation univocally couples with a favorable outcome with vaccination, which can even overcome pre-existing risk factors (older age, comorbidities, anti-IFN-α autoantibodies positivity). Indeed, a prior immunization provides a significant advantage in the race to limit the path towards disease worsening.

## Supplementary Information

Below is the link to the electronic supplementary material.

Supplementary file1 (DOCX 521 KB)

## Data Availability

All data are included in the manuscript.
